# When to Intervene in Renal Masses: Integrating Tumor Biology, Patient Fitness, and Competing Mortality Into Clinical Decision-Making

**DOI:** 10.7759/cureus.112876

**Published:** 2026-07-17

**Authors:** Hasan F Buali, Khalifa Albenjasim, Abdulaziz Al Shaibani, Noora Aljeeran, Mohamed Rafie

**Affiliations:** 1 Urology, King Hamad University Hospital, Busaiteen, BHR

**Keywords:** active surveillance, competing mortality, elderly, frailty, partial nephrectomy, renal cell carcinoma, renal mass, thermal ablation

## Abstract

Renal cell carcinoma is among the most common urologic malignancies worldwide, with the majority of cases now detected incidentally at an early stage. Despite this shift toward earlier detection, clinical decision-making in the management of localized renal masses remains complex, particularly in elderly and comorbid patients. Existing guidelines provide a framework for management but fall short of offering a practical, risk-stratified algorithm for patients in whom competing comorbidities may outweigh the oncologic risk of the mass itself. This narrative review synthesizes current evidence on tumor biology, active surveillance (AS) outcomes, patient fitness assessment, intervention options, and competing mortality to address a central clinical question: when should we intervene in a renal mass, and when should we not? The evidence consistently supports the underuse of AS and the overuse of radical nephrectomy in elderly and high-comorbidity patients. A risk-stratified decision framework integrating tumor size, growth kinetics, frailty, and life expectancy is proposed to guide individualized management.

## Introduction and background

Renal cell carcinoma (RCC) is a common malignancy, with approximately 434,840 incident cases estimated worldwide in 2022, ranking sixth among male cancers and ninth among female cancers in the United States [1]. The majority of patients, approximately 70%, are diagnosed with stage I disease, and an estimated 37% to 61% of cases are detected incidentally on abdominal imaging performed for unrelated indications [1]. This epidemiological shift has created a paradox: while earlier detection carries the theoretical advantage of identifying curable disease, it has also resulted in the widespread treatment of tumors that may never cause harm during the patient's natural lifespan. The rising incidence of incidentally detected renal masses has been accompanied by a parallel increase in the proportion of patients presenting at an advanced age with significant comorbid conditions, creating a clinical population for whom standard treatment algorithms were not designed. Many small renal masses (SRMs) demonstrate indolent biological behavior with low metastatic potential, meaning that a proportion of patients, particularly those of advanced age, may never experience clinical progression from their tumor during their natural lifespan.

Both the American Urological Association (AUA) and European Association of Urology (EAU) have published guidelines on the management of localized renal masses, each endorsing partial nephrectomy (PN) as the preferred approach for clinical T1a lesions and acknowledging active surveillance as an option for selected patients [2,3]. The AUA guideline limits active surveillance to tumors smaller than 2 cm and does not define explicit intervention triggers beyond growth rate and patient preference, while the EAU 2022 update endorses surveillance for selected patients without algorithmizing eligibility criteria for the elderly or frail [2,3]. However, neither guideline provides a usable algorithm for the patient most commonly encountered in clinical practice: the elderly individual with a small renal mass and one or more significant comorbidities. In this patient, the question is not simply whether the mass is malignant, but whether treating it will confer a meaningful survival benefit relative to the risks of intervention. In this population, competing mortality, death from cardiovascular disease, diabetes, or other comorbidities before the renal mass becomes life-threatening, frequently exceeds cancer-specific mortality, fundamentally altering the risk-benefit calculation of intervention.

Literature search and evidence synthesis

PubMed was searched from inception to May 2025 using the following key terms: renal cell carcinoma, renal mass, active surveillance, small renal mass, partial nephrectomy, radical nephrectomy, thermal ablation, frailty, elderly, competing mortality, Charlson comorbidity index, and clinical frailty scale. English-language studies were prioritized. Inclusion priority was given to prospective cohort studies, registry data, randomized controlled trials, and major international guidelines. Narrative reviews and retrospective series were included when higher-level evidence was unavailable for a specific domain. No formal risk-of-bias assessment was performed, consistent with narrative review methodology.

## Review

Tumor biology and growth kinetics

Understanding the natural history of a renal mass is the essential first step in deciding whether to intervene. Not all renal masses behave the same. Clear cell RCC, the most common subtype accounting for 75% to 80% of cases, is characterized by inactivation of the von Hippel-Lindau tumor suppressor gene and carries a more aggressive biology than papillary or chromophobe subtypes [1]. However, at the SRM stage, defined as enhancing solid or complex cystic lesions measuring 4 cm or less, the risk of aggressive behavior is considerably lower than widely assumed.

For cystic renal masses specifically, the Bosniak classification version 2019 provides a structured framework for surveillance. Class III cystic masses demonstrate a median linear growth rate of 0.0 mm per year, with only 7% showing growth exceeding 5 mm per year. Class IV masses, by contrast, grow at a median of 2.3 mm per year, with 28% exceeding the 5 mm per year threshold and a hazard ratio for progression of 5.1 relative to class III lesions [4]. These findings challenge the practice of treating class III and class IV cystic masses uniformly and support a more differentiated surveillance approach.

For solid SRMs, tumor size is the strongest independent predictor of growth rate and the need for delayed intervention. Data from the prospective Delayed Intervention and Surveillance for Small Renal Masses (DISSRM) registry suggest that a 2.9 cm cutoff, derived from maximally selected rank statistics, provides the most meaningful prognostic threshold for predicting growth events exceeding 0.5 cm per year in the DISSRM cohort, though this cutoff has not yet been validated as a universal clinical standard [5]. This has direct implications for counseling: a patient with a 1.5 cm mass can be reassured that significant growth is unlikely, while a patient with a 3.2 cm mass warrants more frequent follow-up and a lower threshold for intervention.

Most SRMs less than 4 cm demonstrate slow growth on surveillance, with very low rates of metastatic progression. Enhanced renal masses below 4 cm have an extremely low metastatic potential, supporting active surveillance (AS) as a legitimate management strategy rather than a deferral of inevitable treatment [6]. Emerging artificial intelligence-based analysis of preoperative CT imaging has demonstrated the ability to predict malignancy with an area under the curve of 0.871 and to differentiate aggressive from indolent tumors with an area under the curve of 0.783, outperforming seasoned radiologists and radiomics models in prospective testing [7]. These tools are not yet standard of care but represent a near-future refinement in the preintervention assessment of renal masses.

Active surveillance: rationale, outcomes, and patient selection

AS of renal masses consists of serial cross-sectional imaging, typically CT or MRI, with deferred treatment reserved for predefined triggers. The rationale rests on three converging bodies of evidence: the indolent natural history of most SRMs, the finite morbidity of surgical intervention, and the growing recognition that many benign masses are resected unnecessarily [8]. Radiology plays a central role, not only in initial characterization but in identifying masses suitable for AS entry and in monitoring for growth, morphologic change, or features suggesting progression to aggressive disease.

The expansion of robotic surgery has outpaced the adoption of AS, creating a situation where SRMs in elderly and comorbid patients are routinely treated with extirpative surgery when surveillance would confer equivalent oncologic outcomes with substantially lower risk [9]. This overtreatment bias is the central gap this review addresses. 

Outcomes from the DISSRM Registry

The DISSRM registry remains the largest and most influential prospective dataset on AS for SRMs. In a cohort of patients 60 years of age or younger, a group in whom concerns about long-term surveillance durability are highest, 30.4% chose AS as their initial management. At a median follow-up of 4.9 years, cancer-specific survival was 100% in both the primary intervention and surveillance groups. No patient developed metastatic disease on AS, and recurrence-free survival at five years was 96.0% and 100% in the primary and delayed intervention groups, respectively [10]. These data suggest that even in younger patients, AS does not compromise oncologic outcomes when appropriately applied, and the implication for older patients with competing mortality risks is even stronger.

Multiple reviews and guidelines have synthesized these findings, consistently supporting AS for selected patients with comorbidities or limited life expectancy [11]. A comprehensive literature and guidelines review across AUA, EAU, ESMO, and other international bodies confirms universal endorsement of AS in this population, while noting variation in how triggering criteria for delayed intervention are defined. 

The Role of Renal Mass Biopsy in Surveillance Decisions

Renal mass biopsy (RMB) has an evolving and increasingly important role in AS decision-making. In a single-center series of 192 biopsies performed for clinical T1a masses, 63% were malignant, 20% were benign, and 17% were nondiagnostic. Following biopsy, 37% of patients elected AS, and the rate of surgery for ultimately benign pathology was reduced to 3% [12]. The AUA recommends biopsy prior to ablation and endorses selective use during AS, while the EAU provides broader support. RMB adds histologic certainty that enables confident surveillance enrollment, particularly in elderly patients in whom avoiding unnecessary intervention carries the greatest value.

Assessing patient fitness: beyond chronological age

Age alone is an inadequate basis for surgical decision-making. Two patients of identical age may have vastly different physiologic reserves depending on their comorbidity burden, functional status, and frailty phenotype. The clinical imperative is to replace chronological age with a structured fitness assessment that predicts postoperative risk accurately. Table 1 summarizes the validated tools most applicable to this patient population.

**Table 1:** Frailty and Fitness Assessment Tools in Urologic Surgery HTN: Hypertension; DM: diabetes mellitus; COPD: chronic obstructive pulmonary disease; ACS-NSQIP: American College of Surgeons National Surgical Quality Improvement Program

Tool	Variables Assessed	Validated in Urology	Clinical Utility
Modified frailty index-5 (mFI-5) [13]	HTN, DM, CHF, COPD, functional status	Yes — ACS-NSQIP urologic surgery database	mFI-5 ≥2 associated with increased in-hospital mortality, sepsis, pneumonia
Clinical frailty scale (CFS) [14]	Mobility, function, disability, dementia (9-point scale)	Yes — urologic malignancy surgery	CFS ≥5 predicts 10x higher major complication risk; 90-day ADL decline
Charlson comorbidity index (CCI)	19 weighted comorbid conditions	Widely used; validated in RCC systemic therapy	Useful for comorbidity burden; does not capture functional reserve
ECOG/KPS [2,3]	Functional performance status (0-4/0-100)	Standard in oncology; included in AUA/EAU guidelines	Simple bedside tool; subjective; does not capture frailty phenotype

The modified frailty index-5 (mFI-5), a five-item composite score assessing hypertension, diabetes, congestive heart failure, chronic obstructive pulmonary disease, and functional status, has been validated in a large propensity-score matched cohort drawn from the American College of Surgeons National Surgical Quality Improvement Program database. Patients with an mFI-5 score of 2 or greater demonstrated significantly higher rates of in-hospital mortality, myocardial infarction, pneumonia, and sepsis following urologic surgery [13].

The clinical frailty scale (CFS) offers a complementary, clinician-administered assessment. In a prospective cohort of 82 patients aged 75 years or older undergoing curative urologic surgery, mean age 81.6 years, a CFS score of 5 or higher was associated with a 10.3-fold higher risk of major complications within 30 days and an 8.5-fold higher risk of activities of daily living decline at 30 days [14]. At 90 days, the risk of functional decline in this group was 21.4-fold higher. These data establish that frailty, not age, is the operative risk predictor, and that patients with CFS scores of 5 or above represent a population for whom surgical intervention carries substantial functional hazard.

The practical implication is straightforward: before listing an elderly patient for nephrectomy, a structured frailty assessment should be performed. An 81-year-old with CFS 3 and a 2 cm mass is a categorically different patient from an 81-year-old with CFS 6 and a 3 cm mass. The former may reasonably tolerate PN or ablation. The latter is best served by AS.

Intervention options: matching treatment to patient and tumor

When the decision to intervene is made, the choice of treatment modality should be driven by tumor size and complexity, functional renal reserve, and patient fitness, not institutional preference or surgeon familiarity alone. Table 2 summarizes the four main management strategies with their candidate profiles, size thresholds, and oncologic outcomes.

**Table 2:** Management Options for Localized Renal Masses by Candidate Profile and Tumor Characteristics

Management Option	Best Candidate	Tumor Size	Oncologic Outcome	Key Limitation
Active surveillance [6,9,10]	Elderly, high comorbidity, limited life expectancy, tumor <2 cm	<2 cm (preferred); up to 4 cm in selected cases	Cancer-specific survival 100% in DISSRM; low metastatic risk	Requires reliable imaging follow-up; patient anxiety
Thermal ablation (RFA/Cryo) [15,16]	Elderly, moderate comorbidity, surgical risk, tumor <2.4 cm	<2.4 cm optimal; up to 3 cm acceptable	5-year RFS 100% for tumors <2.4 cm; shorter stay vs surgery	Higher recurrence in tumors >2.4 cm; local recurrence risk
Partial nephrectomy [17,18]	Fit patients, solitary kidney, bilateral tumors, young age	cT1a-T1b; selected T3a in experienced centers	5-year CSS >94% for <4 cm; preferred nephron-sparing standard	Requires surgical fitness; not suitable for high-frailty patients
Radical nephrectomy [19,20]	Larger tumors, venous involvement, T3a with sinus fat invasion	>4 cm or T3a features	Standard for complex disease; comparable CSS to PN in T3a	Irreversible renal function loss; not ideal for comorbid elderly

Thermal Ablation

Percutaneous thermal ablation, encompassing radiofrequency ablation (RFA) and cryoablation, has emerged as an effective alternative to surgery for SRMs in patients with increased operative risk. A feasibility randomized controlled trial comparing cryoablation to robot-assisted PN demonstrated shorter hospital stay with cryoablation (1 vs 2 days, p = 0.019) and a lower complication rate (12% vs 29%), with comparable six-month renal function decline (-5.0 vs -5.8 mL/min/1.73 m2) [15].

Tumor size is the critical determinant of ablation success. Five-year recurrence-free survival after a single thermal ablation session is 100% for tumors smaller than 2.4 cm, falling to 58.4% for tumors between 2.4 and 4 cm, compared to 98.1% for PN across all sizes [16]. This size-outcome relationship suggests that 2.4 cm, not the conventionally cited 3 cm threshold, may represent a more appropriate cutoff for selecting ablation over PN, based on the findings of this single-institution study [16]. For the elderly or comorbid patient with a mass below 2.4 cm, thermal ablation offers excellent oncologic control with substantially lower procedural burden.

Partial Nephrectomy

PN remains the standard of care for fit patients with T1a and T1b tumors and is the preferred approach for preserving renal function. Robot-assisted PN (RAPN) has extended the applicability of nephron-sparing surgery to increasingly complex tumors. For clinical T3a lesions, including those with perinephric fat, sinus fat, or pelvicalyceal system involvement, RAPN is feasible in experienced centers and does not confer a worse prognosis than radical nephrectomy (RN) when venous involvement is absent [17].

The distinction between imperative and elective indications is clinically relevant. Imperative indications, solitary kidney, bilateral tumors, baseline renal insufficiency, carry higher functional stakes but do not compromise perioperative safety. In a multinational propensity-matched cohort of 3,802 RAPN cases, perioperative outcomes including ischemia time, blood loss, complication rates, and positive surgical margin status were equivalent between imperative and elective groups. The only significant difference was a greater postoperative estimated glomerular filtration rate decline in the imperative group (38.6% vs 11.3%) [18]. These findings suggest that PN is safe for nephron-sparing imperative situations even when baseline function is reduced.

Radical Nephrectomy in the Elderly

RN remains appropriate for larger tumors, venous involvement, or cases where PN is technically not feasible. However, its role in elderly patients with small masses demands scrutiny. In a retrospective series of 672 nephrectomy patients analyzed using optimized age cutoffs, patients aged 75 years or older carried an independent risk for inferior overall survival (hazard ratio 4.36, 95% confidence interval 1.31-14.48), but not for cancer-specific survival [19]. This dissociation between overall and cancer-specific survival is precisely the competing mortality phenomenon: elderly patients die with RCC, not from it.

A separate series of 101 nephrectomy patients comparing outcomes by age below and above 65 years confirmed that operative time, blood loss, and renal function decline were comparable across age groups, but complication rates were 22% versus 12%, and overall survival was shorter in older patients (hazard ratio 2.25, 95% confidence interval 1.08-4.69) [20]. The conclusion shared by both series is that nephrectomy in carefully selected elderly patients is feasible, but that selection, not reflexive surgical referral, is the operative principle. Age alone is not a contraindication, but age combined with frailty and limited life expectancy substantially alters the risk-benefit calculus.

Competing mortality and life expectancy

The concept of competing mortality is central to the management of renal masses in elderly patients. An 81-year-old male with a 3 cm renal mass and significant cardiovascular comorbidity is more likely to die of cardiac disease than of renal cancer. Treating the mass aggressively in this patient may confer surgical risk without meaningful oncologic benefit.

Prognostic models for oncologic outcomes following nephrectomy, developed from the Mayo Clinic nephrectomy registry encompassing 3,633 patients, demonstrate that cancer-specific survival can be predicted with high accuracy from clinicopathologic features. For clear cell RCC, the model achieved a c-index of 0.86 for cancer-specific survival [21]. These models are most useful in identifying patients at genuinely high oncologic risk who warrant aggressive treatment, and by inference, identifying those at low oncologic risk for whom surveillance is appropriate.

The effect of surgical approach on other-cause mortality is also age-dependent. In a propensity-matched SEER database analysis of metastatic RCC patients, noting that this cohort differs from the localized disease population that is the primary focus of this review, PN was associated with significantly lower other-cause mortality in patients under 70 (hazard ratio 0.22 in the under-60 cohort; hazard ratio 0.38 in the 60-69 cohort), but this benefit was absent in patients aged 70 or older [22]. This finding suggests that the renal function-preserving benefit of nephron-sparing surgery, which underlies its association with lower cardiovascular mortality, may diminish with advancing age, further supporting the argument that radical intervention in elderly patients offers less systemic benefit than conventionally assumed.

Comparison of current guidelines

Table 3 summarizes the key differences and points of convergence between the AUA 2017 and EAU 2022 guidelines on localized renal mass management.

**Table 3:** Comparison of AUA 2017 and EAU 2022 Guidelines on Localized Renal Mass Management AUA: American Urological Association; EAU: European Association of Urology

Feature	AUA 2017	EAU 2022	Clinical Implication
Active surveillance indication	Tumors <2 cm; comorbid or elderly patients	Selected patients; criteria less defined	AUA provides a clearer size-based threshold
Trigger for intervention from AS	Defined: growth >5 mm/year, patient preference, size threshold	Not explicitly defined	AUA superior for clinical decision-making
Partial nephrectomy preference	cT1a: strongly preferred; cT1b: recommended when feasible	Recommended for T1 tumors when technically feasible	Both guidelines converge on nephron-sparing priority
Renal mass biopsy	Recommended before ablation; selective use during AS	Recommended before ablation; broader support	Biopsy increasingly standard before non-surgical management
Thermal ablation	Selective use for tumors ≤3 cm; preferred in poor surgical candidates	Alternative for T1a in patients unfit for surgery	Both support ablation as surgery alternative in selected patients
Elderly/comorbid guidance	Acknowledged; individualized counseling recommended	Mentioned but not algorithmized	Gap: neither guideline provides a usable elderly-specific framework

The AUA guideline provides clearer operationalization of AS triggers and biopsy indications but was published in 2017 and does not incorporate more recent DISSRM registry data or the growing evidence base for ablation in elderly patients [2]. The EAU 2022 update offers broader support for biopsy before ablation but lacks a structured algorithm for the elderly or frail patient [3]. Both guidelines converge on the priority of nephron-sparing and the legitimacy of surveillance but leave the clinician without a workable framework for the most challenging cases. This gap is the primary justification for the proposed risk-stratified approach outlined below.

Proposed Decision Framework

In the group of elderly patients with decreased life expectancy, AS is the most appropriate initial approach for SRMs, with focal ablation serving as an intermediate option when the tumor grows beyond the surveillance threshold but the patient remains a poor surgical candidate [23]. This principle, supported by the literature reviewed, allows construction of a practical risk-stratified framework.

The following three-tier approach integrates tumor, patient, and preference factors:

Tier 1 - active surveillance: Tumor below 2 cm, or patient with CFS score 5 or above, or limited life expectancy with competing comorbidities. Serial imaging at three to six monthly intervals in the first year, then annually. Intervention threshold: growth exceeding 5 mm per year, patient preference change, or tumor crossing 3 cm.

Tier 2 - thermal ablation: Tumor between 2 and 2.4 cm with growth documented on surveillance, or patient unfit for general anesthesia but with reasonable functional status (CFS 3 to 4). Preferred modality: cryoablation or RFA. Expected five-year recurrence-free survival exceeds 95% at this size threshold.

Tier 3 - surgical intervention: Tumor exceeding 2.4 cm in a patient with adequate fitness (CFS below 4, mFI-5 below 2). PN is preferred for T1a and T1b lesions. RN is reserved for T3a features, venous involvement, or tumor complexity precluding nephron-sparing. Frailty assessment and shared decision-making are mandatory before proceeding.

This framework acknowledges that no algorithm replaces individualized counseling. The 81-year-old male with a 4 cm mass, CFS 6, ejection fraction of 35%, and stage 3 chronic kidney disease is not a surgical candidate regardless of tumor size. The same patient at CFS 3 with well-controlled hypertension and normal renal function may tolerate ablation or even PN safely. The decision must be built from the patient upward, not from the guideline downward.

## Conclusions

The management of renal masses in elderly and comorbid patients requires a deliberate departure from the default intervention paradigm. The evidence reviewed demonstrates that AS is oncologically safe for most SRMs, thermal ablation provides excellent recurrence-free survival for tumors below 2.4 cm in poor surgical candidates, and RN confers limited cancer-specific survival benefit in patients whose life expectancy is dominated by competing comorbidities. Chronological age is an insufficient basis for surgical decision-making. Structured frailty assessment using validated tools such as the CFS and mFI-5, integrated with tumor biology, growth kinetics, and patient preference, should form the backbone of every treatment decision. Prospective studies comparing active surveillance to ablation in defined elderly cohorts are needed to validate risk-stratified frameworks and fill the gap that current guidelines leave unaddressed.
